# microRNA-150: a promising novel biomarker for hepatitis B virus-related hepatocellular carcinoma

**DOI:** 10.1186/s13000-015-0369-y

**Published:** 2015-07-28

**Authors:** Fujun Yu, Zhongqiu Lu, Bicheng Chen, Peihong Dong, Jianjian Zheng

**Affiliations:** Department of Infectious Diseases, The First Affiliated Hospital of Wenzhou Medical University, No.2 FuXue lane, Wenzhou, 325000 Zhejiang People’s Republic of China; Emergency Department, The First Affiliated Hospital of Wenzhou Medical University, No.2 FuXue lane, Wenzhou, 325000 Zhejiang People’s Republic of China; Key Laboratory of Surgery, The First Affiliated Hospital of Wenzhou Medical University, No.2 FuXue lane, Wenzhou, 325000 People’s Republic of China

**Keywords:** microRNA-150, Hepatocellular carcinoma, Biomarker, Serum

## Abstract

**Background:**

Chronic hepatitis B virus (HBV) infection is a known major etiological factor for hepatocellular carcinoma (HCC) development. Alpha-fetoprotein (AFP) is widely used to detect primary HCC, whereas its sensitivity and specificity are not satisfying. Recently, circulating microRNAs (miRNAs) have been reported to be promising biomarkers for diagnosing and monitoring cancers. This study was conducted to detect the application of serum miR-150 in the diagnosis and prognosis of HBV-related HCC.

**Methods:**

The expression of miR-150 was evaluated using a real-time quantitative RT-PCR in 350 serum samples (120 samples from controls, 110 from chronic hepatitis B (CHB) patients and 120 samples from HCC patients.

**Results:**

Serum miR-150 levels were significantly reduced in HCC patients, compared with healthy controls (*P* < 0.0001) and CHB patients (*P* < 0.0001). Serum miR-150 levels were increased after surgical operation (*P* < 0.0001) and decreased after tumor recurrence (*P* < 0.0001). Receiver operating characteristic curve (ROC) analyses suggested that serum miR-150 had significant diagnostic value for HBV-related HCC. It yielded an area under the curve (AUC) of ROC of 0.931 with 82.5 % sensitivity and 83.7 % specificity in discriminating HCC from healthy controls, and an AUC of ROC of 0.881 with 79.1 % sensitivity and 76.5 % specificity in discriminating HCC from CHB patients. Moreover, Kaplan-Meier curve analysis revealed that HCC patients with lower serum miR-150 had a significantly shortened overall survival (*P* < 0.0001). Univariate and Multivariable Cox regression analysis indicated that serum miR-150 level was an independent risk factor for overall survival (*P* < 0.0001 and *P* = 0.015, respectively).

**Conclusions:**

Serum miR-150 can serve as a non-invasive biomarker for the diagnosis and prognosis of HCC patients.

## Background

Hepatocellular carcinoma (HCC) is one of the most common prevalent cancers, rated third in mortality worldwide [1]. Although there are different viral and non-viral causes of HCC, almost 80 % of HCC patients are associated with hepatitis B virus (HBV) infection [2]. Currently, most HCC patients have a poor prognosis with a relative low survive rate, which is due to their diagnosis at advanced stages with limited therapeutic options. Alpha-fetoprotein (AFP) is widely used to detect primary HCC but its sensitivity and specificity are disputed [3]. Thus, it is prudent to search for more effective and reliable markers for diagnosis and prognosis of primary HCC.

MicroRNAs (miRNAs), a class of small non-coding RNAs, can regulate gene expression by binding to complementary sequences in the 3′-untranslated region of mRNAs [4–6]. Recently, miRNAs have been shown to be involved in several physiological processes such as development, apoptosis, proliferation, and differentiation, and even play a critical role in carcinogenesis [7–9]. In many cancers, aberrant expressions of tissue-miRNAs may lead to poor prognosis, suggesting that they can function as oncogenes or tumor suppressor genes [10, 11]. Apart from their tissue-specific origin and expression, miRNAs are also shown to be stable and detectable in many body fluids including serum and plasma [12]. Increasing evidence has shown that circulating miRNAs have potential as non-invasive biomarker for HCC. For example, Xie *et al.* found that serum miR-101 levels were significantly down-regulated in the HBV-HCC patients and could differentiate HBV-HCC form HBV-associated liver cirrhosis [2]. Notably, altered serum/plasma miRNAs levels are also associated with the development of HCC [13].

Our group previously reported that over-expression of miR-150 contributed to the suppression of activated hepatic stallate cells (HSCs) in liver fibrosis, resulting in the reduction of cell proliferation [14]. Interestingly, Chang *et al*. found that Myc levels could be affected by miR-150 in Myc-mediated tumorigenesis [15]. Zhang *et al*. further demonstrated that miR-150 could inhibit CD133-positive liver cancer stem cells by targeting c-Myb [16]. Combined these, miR-150 might function as a tumour suppressor in HCC. In addition, a recent study showed that miR-150 is a factor of survival in non-small cell lung cancer and associated with poor prognosis [17]. miR-150 also predicts a favorable prognosis in patients with epithelial ovarian cancer, and inhibits cell invasion and metastasis by suppressing transcriptional repressor ZEB1 [18]. In this study, we evaluated whether serum miR-150 could serve as a new biomarker for the diagnosis and prognosis of HBV-related HCC patients.

## Methods

### Study subjects

Serum samples were obtained from patients attending the First Affiliated Hospital of Wenzhou Medical University from 2007 to 2011 (Table 1). This study population consisted of 120 HBV-HCC patients, 110 chronic hepatitis B (CHB) patients and 120 healthy controls (with normal liver biochemistry, no history of liver disease or alcohol abuse and no viral hepatitis). For CHB patients, blood samples were taken at the time when the disease was active. The diagnosis of HCC was based on histopathological assessment [19]. For HCC patients, the pre-operative blood samples were taken at the time of initial consultation. No patients underwent surgery or received chemotherapy or radiotherapy before blood sampling. In this study, HCC patients only underwent surgery and no transplant cases included. Liver resection was performed according to guidelines on the diagnosis and treatment of primary liver cancer in China [20] and the clinical experience of surgeons. In particular, the following requirements should be met for HCC patients with multi-nodular disease or larger nodules: a) Basic conditions: a patient’s general conditions should allow him/her to tolerate surgery; the liver lesions are resectable; and the reserved liver function can serve in compensation. Specifically, a patient in generally good condition should have: no major disorders of the heart, lung, kidney and other vital organs; normal liver function, or with only mild damage (Child-Pugh class A), or once Class B liver function that has recovered to Class A after short-term routine therapy; hepatic functional reserve basically within the normal range; and no unresectable, extrahepatic metastatic lesions; b) Single liver with smooth surface and clearly defined boundaries or pseudocapsule, <30 % liver tissue damaged by tumor, or damaged tissue >30 % but with significantly compensatory enlargement of the contralateral lobes that exceed 50 % or more of the standard liver volume; c) Multiple tumors with less than five nodules that are confined to a single segment or lobe of the liver. The post-operative blood samples were collected a month after surgery and the relapsed blood samples were collected at the diagnosis of tumor recurrence. Only 25 cases of blood samples could be collected from the relapsed patients. Among these patients, 5 cases with tumor metastasis are confirmed. Notably, none underwent surgery or received chemotherapy or radiotherapy before blood sampling. In addition, Child-Pugh score [21] and the Barcelona Clinic Liver Cancer (BCLC) stage [22] were assessed by results of clinical examination, imaging (dynamic computer tomography, magnetic resonance imaging or abdominal ultrasound examination) and laboratory parameters. The follow-up period is used for calculating HCC patients’ survival rate. The follow-up period was defined as the time from the date of surgery to the date of patient mortality or the last follow-up point. Follow up was completed on January 1, 2013. All HCC patients were monitored after surgery; diagnosis of recurrence was confirmed by ultrasound, enhanced CT scan, MRI, and AFP levels. This project was approved by the Ethics Committee of the First Affiliated Hospital of Wenzhou Medical University. Informed consent was obtained from all participants for the use of their blood samples in this study.

**Table 1:** Patient information

Characteristic	HCC (*n* = 120)	CHB (*n* = 110)	Control (*n* = 120)
Gender			
Male	75	67	65
Female	45	43	55
Age			
Mean ± SD	58 ± 10.4	55 ± 11.2	50 ± 9.5
AFP value			
Mean ± SD	293.7 ± 705.5	18.8 ± 16.5	3.1 ± 1.2
HBsAg status			
HBsAg^+^	120	110	0
HBsAg^-^	0	0	120
Child-Pugh stage			
A	81	95	
B	39	15	
Tumor diameter (cm)			
≥5	42		
<5	78		
Differentiation			
Poor	59		
Moderate + Well	61		
TNM stages			
I + II	73		
III	47		
BCLC stage			
A	70		
B	50		

*HCC* hepatocellular carcinoma; *CHB* chronic hepatitis B

### Samples processing and RNA extraction

The blood samples from all subjects were centrifuged at 3400 g for 7 min at room temperature, and the supernatants were transferred into Eppendorf tubes followed by further centrifugation at 12000 g for 10 min at 4 °C. Then the supernatants were stored at -80 °C pending RNA extraction. All blood samples were processed within 4 h after they were obtained. Total RNA containing small RNA was extracted from 500 μl of serum using a miRNeasy Mini Kit (Qiagen, Carlsbad, California, USA) according to the manufacturer’s instruction for liquid samples. DNase treatment (Qiagen, Carlsbad, California, USA) was carried out to remove any containing DNA. The final elution volume was 20 μl. All serum RNA preparations were quantified by NanoDrop 1000 (Nanodrop, Wilmingtion, Delaware, USA).

### microRNA quantification by real-time quantitative RT-PCR

Serum miR-150 level was quantified in triplicate by qRT-PCR using TaqMan MicroRNA Assay Kits (Applied Biosystems, Foster City, CA). The reverse transcription reaction was performed in a 20 μl reaction volume using specific primer for miR-150 contained in the TaqMan MicroRNA Reverse Transcription kit (Applied Biosystems, Foster City, CA). For synthesis of cDNA, the reaction mixtures were sequentially incubated at 16 °C for 30 min, 42 °C for 30 min, and 85 °C for 5 min. According to the standard TaqMan MicroRNA assay protocol, real-time PCR was performed in ABI 7500 Real-Time PCR system (Applied Biosystems, Foster City, CA) with the following cycle: 95 °C for 10 min, followed by 40 cycles of 95 °C for 15 s and 60 °C for 60 s. Each PCR mixture (20 μl) included the reverse transcription products, TaqMan 2X Universal PCR Master Mix without UNG Amperase, miRNA-specific TaqMan probes, and primers supplied by Applied Biosystems. The cyclethreshold (Ct) values were calculated with the SDS 2.0.1 software (Applied Biosystems, Foster City, CA). The formula 2^−⊿Ct^ was used to calculate the miRNA levels in serum, where ⊿Ct = mean (Ct of internal references) − Ct of target miRNA. The relative expression levels of miR-150 were calculated and normalized to miR-16 (Applied Biosystems, Foster City, CA) using the comparative⊿Ct method and the equation 2 ^−⊿Ct^, as described previously [23].

### Statistical analysis

ANOVA and *χ*^2^ test were used to compare demographic characterization of study population. The significance of serum miR-150 levels was determined by Mann–Whitney *U* test. All tests were two-sided test and *P* < 0.05 was considered as statistically significant. Receiver operating characteristic (ROC) curves were generated to classify patients in different groups, as well as for the evaluation of the diagnostic potential of serum miR-150 via calculation of the area under the ROC curve (AUC), sensitivity and specificity according to standard formulas. Survival curves were plotted using the Kaplan–Meier method and analyzed using the log-rank test. Univariate and multivariate analyses of HCC prognostic factors were performed using the Cox proportional hazards model. Statistical analyses were performed with SPSS 13.0 (IBM, Armonk, NY).

## Results

### Patient population

Demographic and clinical characterizations of study population are summarized in Table 1. A total of 350 participants including 120 HCC patients, 110 CHB patients and 120 healthy controls were recruited into this study. There were no significant differences of age between patients with HCC patients, CHB patients and healthy controls (*P* = 0.562, ANOVA). The sex distribution in the HCC group was 75:45, in CHB group was 67:43 and in control group was 65:55 (*P* = 0.381, *χ*^2^ test).

### Expression profile of serum miR-150

**Fig. 1:**
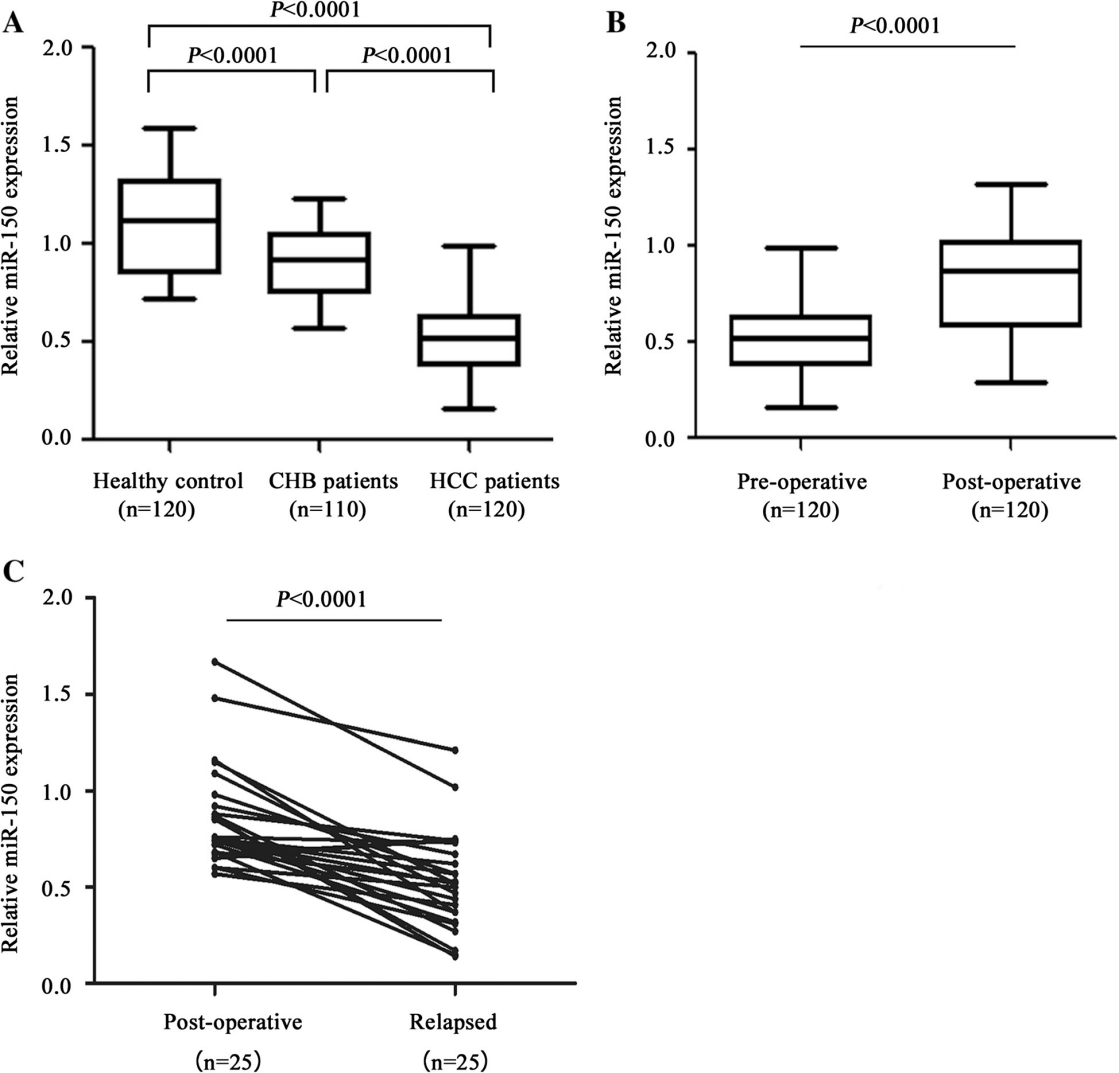
Serum miR-150 levels in healthy controls (n = 120), CHB patients (*n* = 110), HCC patients including pre-operative and post-operative (*n* = 120), and relapsed patients (*n* = 25). **a** Comparison of miR-150 levels among the healthy control, CHB group, and HCC group (pre-operative). **b** Comparison of miR-150 levels between pre-operative group and post-operative group. **c** Comparison of miR-150 levels between paired post-operative and relapsed serums. The lines represent the range and median of relative miR-150 expression

Using qRT-PCR analysis, serum miR-150 expression levels were measured in HCC patients, CHB patients and healthy controls. Serum miR-150 levels were significantly reduced in CHB patients group compared with healthy control (*P* < 0.0001) (Fig. 1a). In addition, serum miR-150 levels were significantly down-regulated in HCC patients compared with the CHB patients (*P* < 0.0001) and healthy controls (*P* < 0.0001) (Fig. 1a). Notably, the reduced serum miR-150 levels were restored after surgical operation. Serum miR-150 levels were significantly up-regulated in post-operative group compared with pre-operative group (*P* < 0.0001) (Fig. 1b). To further verify whether serum miR-150 level is associated with HCC progression, serum miR-150 levels were analyzed by qRT-PCR in 25 paired post-operative and relapsed serum samples. We found that miR-150 levels were significantly down-regulated in the relapsed samples compared with the post-operative samples (*P* < 0.0001) (Fig. 1c).

### The diagnostic value of serum miR-150 for HCC

**Fig. 2:**
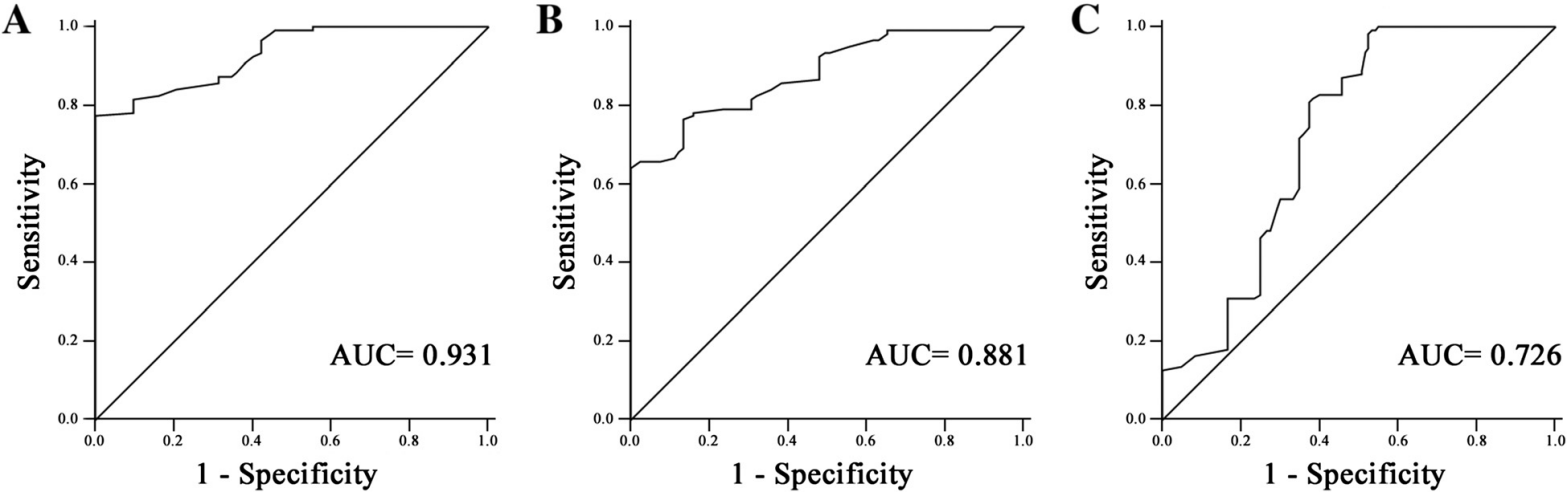
ROC curve analysis of serum miR-150 for discriminating; (**a**) HCC patients from healthy controls, (**b**) HCC patients from CHB patients, (**c**) CHB patients from healthy controls

To confirm whether serum miR-150 level could be served as a potential diagnostic marker for HCC, ROC curve analysis was performed. Our results suggested that serum miR-150 levels differentiated HCC patients from healthy controls, with an AUC of ROC curve of 0.931 [95 % confidence interval (CI), 0.900 to 0.962] (Fig. 2a). At the cutoff value of 0.720, the sensitivity and the specificity were 82.5 % and 83.7 %, respectively. Next, we examined whether serum miR-150 levels could differentiate HCC patients from CHB patients. The results indicated that serum miR-150 levels differentiated HCC patients from CHB patients, with an AUC of ROC curve of 0.881 (95 % CI 0.837 to 0.926) (Fig. 2b). At the cutoff value of 0.650, the sensitivity and the specificity were 79.1 % and 76.5 %, respectively. The potential diagnostic value of miR-150 for differentiating CHB patients with healthy control was additionally evaluated. However, ROC curve analyses showed that the AUC of serum miR-150 level for discriminating CHB patients from healthy controls was only 0.726 (95 % CI 0.660 to 0.793) (Fig. 2c). At the cut-off value of 1.01, the sensitivity was 71.8 %, and the specificity was 65.0 %.

### Serum miR-150 level, clinical characteristics, liver function and survival in HCC patients

**Fig. 3:**
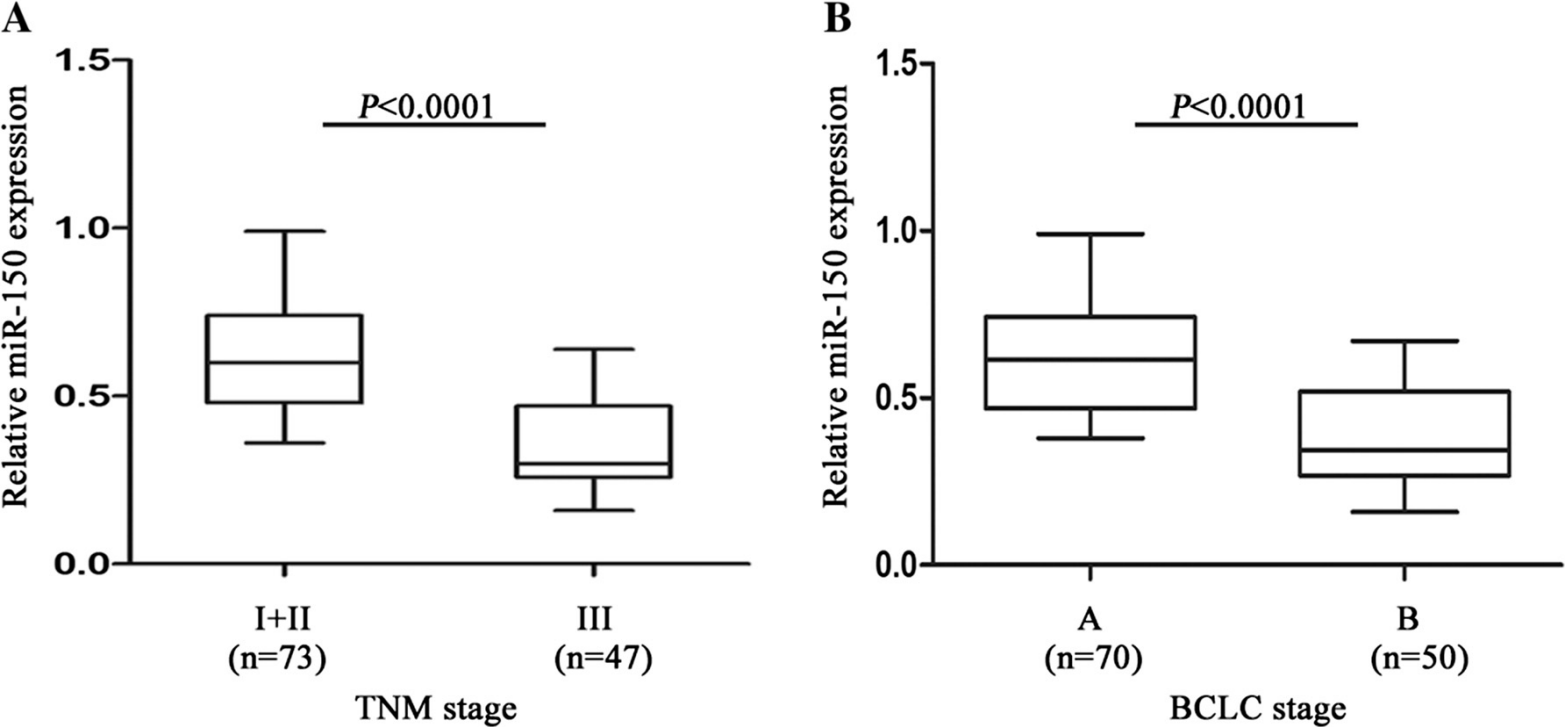
Serum miR-150 levels in HCC patients with different TNM stages or BCLC stages. **a** The levels of miR-150 in TNM stages. I + II represented TNM I and TNM II stages, and III represented TNM III stage. **b** The levels of miR-150 in BCLC stages

**Fig. 4:**
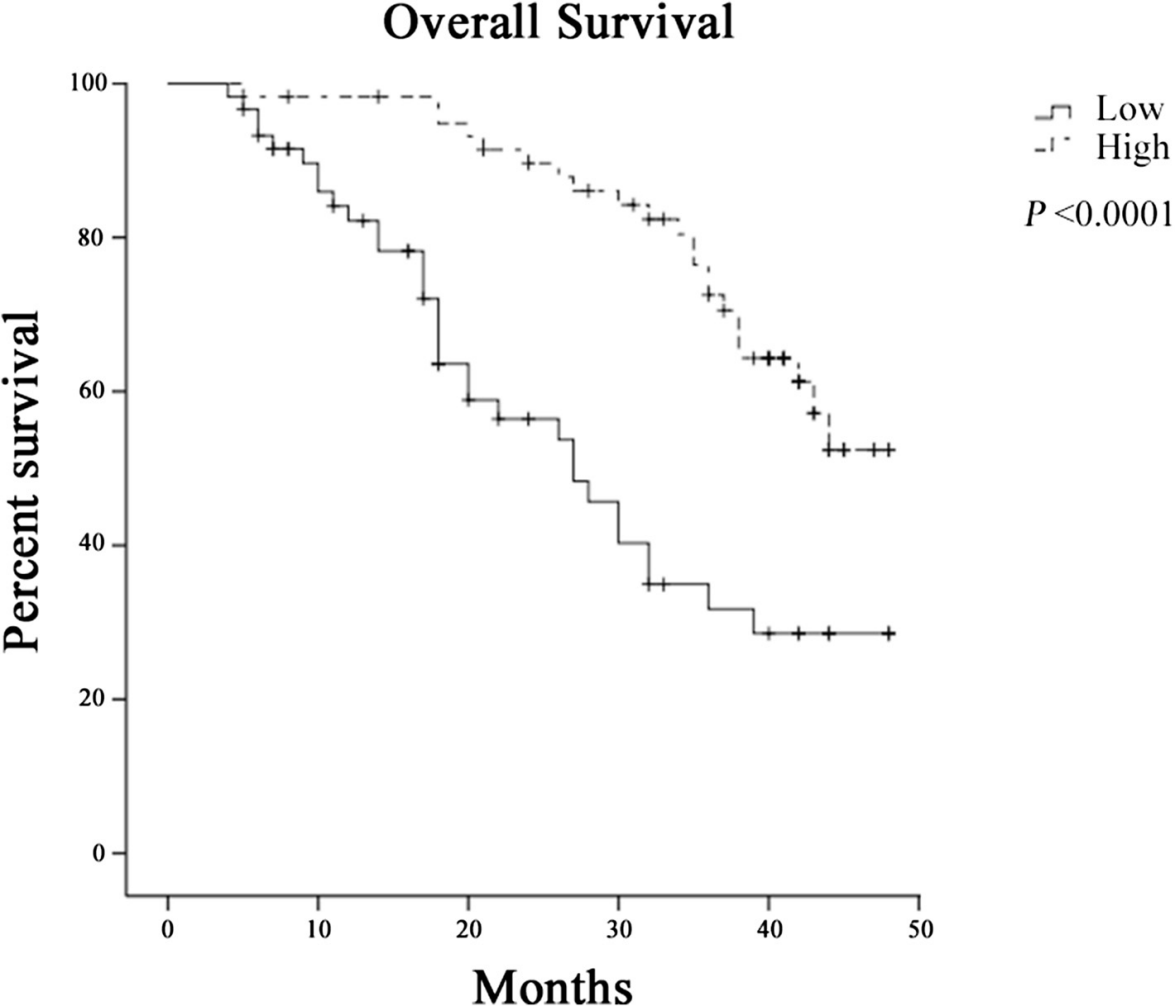
Kaplan–Meier survival curves for HCC patients with low or high expression of serum miR-150

**Fig. 5:**
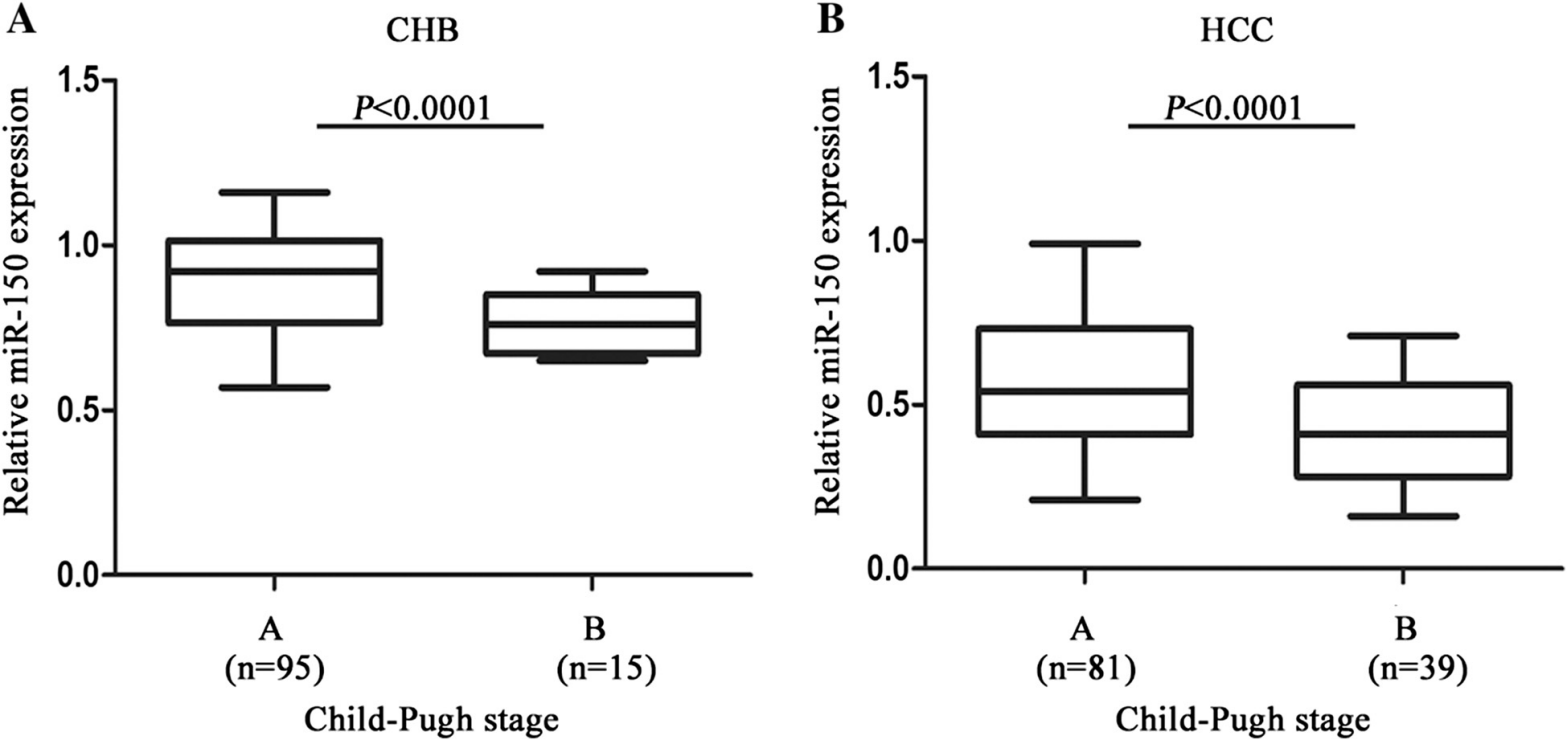
Serum miR-150 levels in patients with different Child-Pugh stages. **a** The levels of miR-150 in CHB patients with different Child-Pugh stages. **b** The levels of miR-150 in HCC patients with different Child-Pugh stages

According to miR-150 median value (0.520), we divided the pre-operative serum samples into low expression and high expression groups in combination with the clinicopathological data. The association between miR-150 expression and clinical parameters was analyzed (Table 2). We found that serum miR-150 levels were significantly associated with TNM and BCLC stages (*P* < 0.0001) (Fig. 3), while there was no significant association of miR-150 with clinical features such as gender, age, AFP levels, tumor diameter and differentiation (*P* > 0.05). Furthermore, the association between miR-150 expression and prognosis was analyzed using Kaplan-Meier analysis and log-rank test. The results suggested that HCC patients with high expression of serum miR-150 had a higher survival rate than those with low miR-150 expression (median overall survival, 40.7 months *vs* 28.3 months, *P* < 0.0001) (Fig. 4). Univariate and multivariate Cox regression models were used to confirm the variables of potential prognostic significance in all HCC patients (Table 3). The result of univariate Cox regression analysis indicated that the reduced levels of serum miR-150 were significantly correlated with overall survival [relative risk (RR), 0.329; *P* < 0.0001]. The multivariate Cox regression analysis demonstrated that serum miR-150 level was an independent prognostic factor for overall survival [RR, 0.446; *P* = 0.015]. As confirmed by multivariate Cox regression analysis, BCLC and TNM stages were also independent prognostic factor for overall survival [RR, 2.631; *P* = 0.006 and RR, 2.180; *P* = 0.020, respectively]. Moreover, to investigate whether miR-150 level is related to liver function, we assessed Child-Pugh scores in CHB and HCC patients. It was found that miR-150 levels in CHB patients with Child-Pugh A stage were higher than that of Child-Pugh B stage (*P* < 0.0001) (Fig. 5). Likewise, higher miR-150 levels were found in HCC patients with Child-Pugh A stage compared with patients with Child-Pugh B stage (*P* < 0.0001) (Fig. 5).

**Table 2:** Correlation of serum miR-150 with clinicopathological data in pre-operative group

Parameter	Number of cases	miR-150 expression		
		Low	High	P-value
Gender				
Male	75	39	36	0.866^a^
Female	45	21	24	
Age (years)				
≥60	64	32	32	0.315^a^
<60	56	28	28	
AFP (ng/ml)				
≥20	88	48	40	0.281^a^
<20	32	12	20	
Tumor diameter (cm)				
≥5	42	26	16	0.174^a^
<5	78	34	44	
Differentiation				
Poor	59	27	32	0.546^a^
Moderate + Well	61	33	28	
BCLC stage				
A	70	20	50	<0.0001^a,b^
B	50	40	10	
TNM stages				
I + II	73	22	51	<0.0001^a,b^
III	47	38	9	

^a^Mann-Whitney *U* test; ^b^ *P* < 0.05

**Table 3:** Univariate and multivariate analysis for the prognostic significance of clinicopathological characteristics and serum miR-150 levels in HCC

Parameter	Univariate analysis		Multivariate analysis^a^	
	HR (95 % CI)^b^	P-value	HR (95 % CI)^b^	P-value
Gender^c^	0.638 (0.358–1.136)	0.127		
Age^c^	0.892 (0.520–1.528)	0.677		
AFP^c^	1.693 (0.903–3.173)	0.095		
Tumor diameter^c^	1.567 (0.909–2.703)	0.106		
Differentiation^c^	1.560 (0.908–2.679)	0.107		
BCLC stages^c^	3.284 (1.830–5.540)	<0.001^d^	2.631 (1.309–5.286)	0.006^d^
TNM stages^c^	3.133 (1.803–5.444)	<0.001^d^	2.180 (1.129–4.210)	0.020^d^
miR-150	0.329 (0.189–0.571)	<0.001^d^	0.446 (0.233–0.854)	0.015^d^

^a^Backward Wald test used for variables screened, *P* = 0.05 was chosen as a criteria for significance; ^b^CI, 95 % confidence interval; ^c^gender, male vs. female; age, ≥60 vs. <60 years; AFP, ≥20 vs. <20 ng/ml; tumor diameter, ≥5 vs. <5 cm; differentiation, poor vs. moderate and well; BCLC stage, B vs. A; TNM stage, III vs. I and II; ^d^ *P* < 0.05

## Discussion

HCC is the leading cause of cancer mortality in many countries due to its high mortality rate [24]. Screening for HCC allows early-stage diagnosis of the malignancy and potentially reduces mortality of the disease. Current diagnosis of HCC is based on imaging technology, serum AFP levels, and histology [25]. These diagnostic tools have variable effectiveness for early diagnosis of HCC; cross-sectional imaging typically detects only tumors greater than 1 cm in diameter, and serologic studies also lack sensitivity and specificity in patients with small tumors [26]. Based on these, investigators are attempting to search for more effective serum biomarker in HCC patients.

In the last decades, numerous studies have shown that aberrant miRNAs expression is associated with the development and progression of various types of human cancer, which indicates that miRNAs can be reliable biomarkers for cancers [27–29]. Tissue-specific miRNAs cannot be used on a wide scale because the procedure is invasive; however, use of serum miRNAs is noninvasive and thus more practical. Notably, serum miRNAs are remarkably stable and expression patterns seem to be tissue-specific, which makes it a good candidate for noninvasive cancer testing [26]. The serum miRNAs were firstly detected in the patients with diffuse large B cell lymphoma, and subsequent studies have continually reported the presence of miRNAs in circulation system and body fluid, and revealed that miRNAs are potential diagnostic biomarkers and prognostic factors in cancers [12, 24, 30–32]. More recently, it was reported that plasma miR-155, miR-197, and miR-182 could be potential noninvasive biomarkers for early detection of lung cancer [32]. Li *et al*. identified miR-18a as a potential marker for hepatitis B virus-related HCC Screening [24]. Huang *et al.* found that plasma miR-29a and miR-92a have significant diagnostic value in advanced neoplasia [33]. These reports prompted us to reveal more useful circulating miRNA markers for different types of cancer with a clinically satisfactory degree of sensitivity and specificity.

In the present study, it was found that serum miR-150 levels were significantly reduced in patients with HBV-related HCC when compared to those in healthy controls, which was consistent with the previous studies that indicated the reduction of miR-150 levels in HCC tissue and cell lines [34, 35]. It yielded an AUC of 0.931, with the sensitivity of 82.5 % and the specificity of 83.7 %. Chronic HBV infection is a known major etiological factor for HCC development [36]. Therefore, we further determine whether serum miR-150 could discriminate HCC patients from CHB patients. Our results showed that serum miR-150 could discriminate HCC patients from CHB patients. It yielded an AUC of 0.881, with the sensitivity of 79.1 % and the specificity of 76.5 %. In addition, serum miR-150 levels were measured in 120 paired pre-operative and post-operative samples. We found that serum miR-150 levels were increased in patients with HCC after surgical resection of tumors. However, serum miR-150 levels were reduced again after tumor recurrence in paired post-operative and relapsed samples. These results suggested that miR-150 acts as a tumor suppressor in the development of HCC. The prediction of metastasis, recurrence, and prognosis in patients with HCC after hepatic resection is an important clinical issue that could determine the surgical therapeutic regimen. In this study, we observed that reduced miR-150 expression in HCC patients was correlated negatively with advanced TNM stages, which is highly correlated with the prognosis of HCC [37, 38]. These data showed that miR-150 could be used for the prediction of the prognosis of HCC. Consistent with this result, further study indicated that the down-regulated miR-150 was associated with poor survival for patients with HCC. The patients with low expression of serum miR-150 had lower survival rates. To our knowledge, it is the first report to evaluate the prognosis value of serum miR-150 in HCC. Moreover, the univariate and multivariate analysis with Cox regression models revealed that serum miR-150 could potentially serve as an independent risk predictor for the prognosis of HCC. Further study showed that miR-150 levels were reduced in CHB patients with Child-Pugh B compared with patients with Child-Pugh A. The similar results are also shown in HCC patients, suggesting that miR-150 level is associated with liver function.

The prediction of the prognosis and accurate patient stratification are crucial to optimise personalised treatment [39]. Besides TNM stages, BCLC staging system is also an often used clinical classification for HCC patients [22, 40]. Our results showed that lower miR-150 level was found in patients with BCLC B stage compared with patients with BCLC A stage, suggesting that miR-150 is also associated with BCLC stage. Recently, it has been demonstrated that the multikinase inhibitor sorafenib has been validated to treat patients with advanced HCC [41]. However, there is no advanced BCLC stage such as BCLC C stage, due to the reason that none with metastasis were included in this study. Our results showed that miR-150 might be a tumor suppressor. Therefore, it is speculated that miR-150 might be associated with tumor metastasis. Whether miR-150 could be tested as a biomarker for sorafenib efficacy should be studied in future. In addition, there are other limitations in this study. First, the sample size is relatively small and large samples are needed to the further validations of this marker. Second, where serum miRNAs come from or how organs release miRNAs into the blood is still unknown. Further studies are needed to prove it.

## Conclusion

In conclusion, our findings suggested that serum miR-150 might serve as a novel diagnostic and prognostic marker for HCC. Our data serve as basis for further investigation, preferably in large prospective studies before miR-150 can be used as a noninvasive screening tool for HCC in routine clinical practice.
